# Facilitators and barriers to compliance with COVID-19 guidelines: a structural topic modelling analysis of free-text data from 17,500 UK adults

**DOI:** 10.1186/s12889-021-12372-6

**Published:** 2022-01-06

**Authors:** Liam Wright, Elise Paul, Andrew Steptoe, Daisy Fancourt

**Affiliations:** 1 Institute of Education, University College London, 55-59 Gordon Square, London, WC1H 0NU UK; 2 Department of Behavioural Science and Health, University College London, London, UK 1-19 Torrington Place, WC1E 7HB

**Keywords:** COVID-19, Compliance, Adherence, Non-pharmaceutical interventions, Free-text data, Structural topic modelling, Text mining

## Abstract

**Background:**

During the COVID-19 pandemic, the UK government implemented a series of guidelines, rules, and restrictions to change citizens’ behaviour to tackle the spread of the virus, such as the promotion of face masks and the imposition of lockdown stay-at-home orders. The success of such measures requires active co-operation on the part of citizens, but compliance was not complete. Detailed research is required on the factors that aided or hindered compliance with these measures.

**Methods:**

To understand the facilitators and barriers to compliance with COVID-19 guidelines, we used structural topic modelling, a text mining technique, to extract themes from over 26,000 free-text survey responses from 17,500 UK adults, collected between 17 November and 23 December 2020.

**Results:**

The main factors facilitating compliance were desires to reduce risk to oneself and one’s family and friends and to, a lesser extent, the general public. Also of importance were a desire to return to normality, the availability of activities and technological means to contact family and friends, and the ability to work from home. Identified barriers were difficulties maintaining social distancing in public (due to the actions of other people or environmental constraints), the need to provide or receive support from family and friends, social isolation, missing loved ones, and mental health impacts, perceiving the risks as low, social pressure to not comply, and difficulties understanding and keep abreast of changing rules. Several of the barriers and facilitators raised were related to participant characteristics. Notably, women were more likely to discuss needing to provide or receive mental health support from friends and family.

**Conclusion:**

The results demonstrated an array of factors contributed to compliance with guidelines. Of particular policy importance, the results suggest that government communication that emphasizes the potential risks of the virus and provides simple, consistent guidance on how to reduce the spread of the virus would improve compliance with preventive behaviours as COVID-19 continues and for future pandemics.

**Supplementary Information:**

The online version contains supplementary material available at 10.1186/s12889-021-12372-6.

## Background

To tackle the COVID-19 pandemic, governments focused on reducing transmission of the virus by influencing citizens’ behaviour. Governments mandated the wearing of face masks in public places, recommended social distancing, promoted regular handwashing, and even ordered the closing of businesses, prohibited household mixing, and implemented stay-at-home “lockdown” orders. These measures (where followed) are effective at reducing infection rates [1]. But while compliance was high overall, it was not complete [2, 3], and levels of compliance in general decreased over the course of the pandemic [4, 5]. Promoting compliance with preventive behaviours was an important component of efforts to tackle the pandemic, particularly prior to the development of a vaccine. For example, in the UK, some measures were backed with the force of law, with individuals breaking travel or household mixing rules subject to fines. Public health messaging was also widely used, and emphasised the need to save lives and protect the National Health Service (NHS).

Policymakers’ beliefs about compliance behaviour – the extent to which citizens comply, for how long, and in which contexts – influenced the measures that have been put in place. For instance, the possibility of “risk compensation” [6] – individuals offsetting one risk-reducing behaviour with riskier behaviours elsewhere – was central to debates on making face masks compulsory in public places [7]. More controversially, the possibility of (behavioural) “fatigue” was cited as a reason to delay lockdown in the UK, with fears that citizens would not sustain compliance over a sufficiently long period of time [8]. Although this argument was widely criticised by behavioural scientists as lacking clarity and scientific support [9–11], it has received limited direct empirical testing to date [4, 5, 12].

Given the importance of citizens’ behaviour for tackling COVID-19 – and previous – pandemics, a large literature has grown on the determinants of compliance, including on the motivations, barriers, and facilitators of compliance and the personal and situational characteristics associated with high compliance levels (for reviews, see [13–15]). Recent work has shown a role of gender [14], worries about the virus [16, 17], pro-social motivations [16, 18], attitudes to risk [19], and assorted personality traits [20, 21] in predicting compliance during COVID-19. However, an issue with the literature on the determinants of compliance is that most studies use quantitative data. A limitation of this is that the factors studied are restricted to those the researcher has thought of in advance. Moreover, in many of these studies, the specific reasons why the studied factors were related to compliance has not been empirically examined. For instance, several studies have shown a link between confidence or trust in government and compliance behaviour [22–24], but the specific barriers or motivations to compliance that may be influenced by trust have not been explored. This limits the lessons that can be drawn.

Qualitative studies offer an opportunity to deepen our understanding of compliance behaviour, allowing for greater flexibility in the exploration and identification of relevant phenomena. Recent qualitative studies from the UK found evidence of “alert fatigue”, with individuals expressing difficulty keeping abreast of frequently changing guidelines and, as a result, inadvertently breaking (or knowingly bending) government rules [25, 26]. It is difficult to imagine a purely quantitative study that would have identified these phenomena. Nevertheless, the small sample sizes typical of qualitative studies also limit the questions that can be asked – notably, those that statistically relate participants’ characteristics to the themes they discuss.

Text-mining approaches offer a middle ground, allowing for the extraction of themes from large-scale free-text data that can then be related to document metadata, such as the date the text was written and the characteristics of its author (e.g., their age, sex, or personality traits). Participants can provide spontaneous information, which can be summarised quantitatively and analysed as any other quantitative variable (e.g., in a regression model). In this study, we used structural topic modelling (STM) [27] – a text-mining technique – to identify facilitators and barriers to compliance from free-text responses from over 17,500 UK adults during the COVID-19 pandemic, relating these to participants’ demographic, socioeconomic and personality characteristics identified as predictors of compliance behaviour in the wider compliance literature.

## Methods

### Participants

We used data from the COVID-19 Social Study; a large panel study of the psychological and social experiences of over 70,000 adults (aged 18+) in the UK during the COVID-19 pandemic. The study commenced on 21 March 2020 and involved online weekly data collection for 22 weeks with monthly data collection thereafter. Data collection is still ongoing. The study is not random and therefore is not representative of the UK population, but it does contain a heterogeneous sample. The sample was recruited using three primary approaches. First, convenience sampling was used, including promoting the study through existing networks and mailing lists (including large databases of adults who had previously consented to be involved in health research across the UK), print and digital media coverage, and social media. Second, more targeted recruitment was undertaken focusing on (i) individuals from a low-income background, (ii) individuals with no or few educational qualifications, and (iii) individuals who were unemployed. Third, the study was promoted via partnerships with third sector organisations to vulnerable groups, including adults with pre-existing mental health conditions, older adults, carers, and people experiencing domestic violence or abuse. Full details on sampling, recruitment, data collection, data cleaning and sample demographics are available at https://github.com/UCL-BSH/CSSUserGuide. The study was approved by the UCL Research Ethics Committee [12,467/005] and all participants gave informed consent.

A one-off module on compliance behaviour was included in the survey between 17 November and 23 December 2020. The module included two free-text questions on facilitators and barriers to compliance, respectively:

1. Since the pandemic started, what has been encouraging or helping you to follow the guidelines, even if only to a partial extent?
2. If you have not been entirely following the guidelines, what are the factors that have been hindering you or acting as obstacles?

We conducted analyses for both questions, separately. Twenty-five thousand fifty-one individuals participated in the data collection containing the free-text survey module (34% of participants with data collection by 23 December 2020). Responses to the free-text questions were optional. Eighteen thousand seven hundred forty-two participants provided a response to the question on facilitators of compliance (74.82% of eligible participants) and 11,902 participants recorded a response to the question on barriers (47.51% of eligible participants). Of these, 16,512 (88.1%) and 9720 (81.6%) participants provided a valid record, the definition of which is provided below.

The period 17 November – 23 December 2020 overlapped with the beginning of the second wave in the UK and the announcement, and start of the rollout, of a vaccine against COVID-19. Government rules changed across the study period. A description of the changes in these rules is provided in the Supplementary Information.

### Data cleaning

We performed topic modelling using unigrams (single words). Responses were cleaned using an iterative process. We excluded responses containing fewer than five words and removed words that appeared in fewer than five responses. We also removed common “stop” words (“the”, “and”, “I”, etc.) from the analysis. We used complete case data so excluded a small number of participants with missing data on any covariate used in the analysis. Spelling mistakes were identified with the hunspell algorithm [28], amended manually if they had five or more occurrences, and replaced using the hunspell suggested word function if the number of occurrences was fewer than five. Where the algorithm provided multiple suggestions, the word with the highest frequency in the original dataset was used. We concatenated frequently-used multi-word concepts into single phrases where we deemed this to be important to our research question (e.g., “high risk”, “pre pandemic”). To reduce data sparsity, in the structural topic models, we used word stemming using the Porter [29] algorithm. Data cleaning was carried out in R version 3.6.3 [30].

### Data analysis

We performed several quantitative analyses. First, as not all participants chose to provide a response, we ran a logistic regression model to explore the predictors of providing a free-text response. Second, we used STM, implemented with the stm R package [31], to extract topics from responses, with the analysis carried for each question separately. STM treats documents as a probabilistic mixture of topics and topics as a probabilistic mixture of words. It is a “bag of words” approach that uses correlations between word frequencies within documents to define topics. STM allows for inclusion of covariates in the estimation model, such that the estimated proportion of a text devoted to a topic can differ according to document metadata (e.g., characteristics of its author). We included participant’s gender, ethnicity (white, non-white), age (modelled with basis splines [B-Splines] with four degrees of freedom [32] to account for potential non-linear association), education level (degree or above, A-Level or equivalent, GCSE or below), living arrangement (alone; not alone, without child; not alone, with child), psychiatric diagnosis (any vs. none), long-term physical health conditions (0, 1, 2+), self-isolation status (yes vs no), and Big-5 personality traits (Openness to Experience, Conscientiousness, Extraversion, Agreeableness, Neuroticism; BFI-2 [33]), each collected at first data collection, and confidence in government to handle the pandemic (1 “None at all” – 7 “Lots”) collected during the same data collection as the free-text responses. For confidence in government, participants from devolved nations were asked about their home government, while those from England were asked about the central UK government. There was only a small amount of item missingness, so we used complete case data. More detail on the variables used in this analysis is provided in the Supplementary Information.

We ran STM models from 2 to 30 topics for each question and selected the final models based on visual inspection of the semantic coherence and exclusivity of the topics and close reading of exemplar documents representative of each topic. Semantic coherence measures the degree to which high probability words within a topic co-occur, while exclusivity measures the extent to that a topic’s high probability words have low probability for other topics. After selecting a final model, we carried out two further analyses. First, we decided upon narrative descriptions for the topics based on high probability words, high “FREX” words (a weighted measure of word frequency and exclusivity), and exemplar texts (responses with a higher proportion of text estimated for a given topic). Second, we ran regression models estimating whether topic proportions were related to author characteristics defined above. Third, we ran a regression model estimating whether self-reported adherence to COVID-19 guidelines (Are you following the recommendations from authorities to prevent spread of Covid-19? 1 “Not at all” - 7 “Very much so”) was related to topic proportions, to explore which barriers to compliance may make compliance particularly challenging.

Data cleaning and analysis was carried out by LW. LW and EP selected the number of model topics and LW, EP, AS and DF agreed upon narrative titles for the topics. The datasets generated and/or analysed during the current study are not publicly available due stipulations set out by the ethics committee but are available from the corresponding author on reasonable request. The code used in the analysis is available at https://osf.io/nf4m9/.

### Role of the funding source

The funders had no final role in the study design; in the collection, analysis, and interpretation of data; in the writing of the report; or in the decision to submit the paper for publication. All researchers listed as authors are independent from the funders and all final decisions about the research were taken by the investigators and were unrestricted.

## Results

### Descriptive statistics

Sixteen thousand five hundred twelve participants provided a valid free-text response to the question on facilitators, and 9720 individuals provided a valid free-text response to the question on barriers (17,706 unique individuals overall). The median response length for the valid free-text responses was 18 (IQR = 10, 33; SD = 33.2) for the question on facilitators and 22 (IQR = 12, 41; SD = 38.5) for the question on barriers. Descriptive statistics for respondents are displayed in Table S1, with figures for the total sample also shown for comparison. Participants in the COVID-19 Social Study are disproportionately female, of older age, and more highly educated, relative to the general population [34]. There were some differences between those who provided a (valid) response and those that did not. Figure S1 displays the results of logistic regression models exploring the predictors of providing a response. Responders to the question on facilitators had higher than average levels of compliance with COVID-19 guidelines, while responders to the question on barriers had lower than average compliance levels. Responders for both questions were disproportionately female, more highly educated and had lower confidence in government, on average. There were also differences according to health and personality traits, though the direction of the association differed by question.

**Fig. 1:**
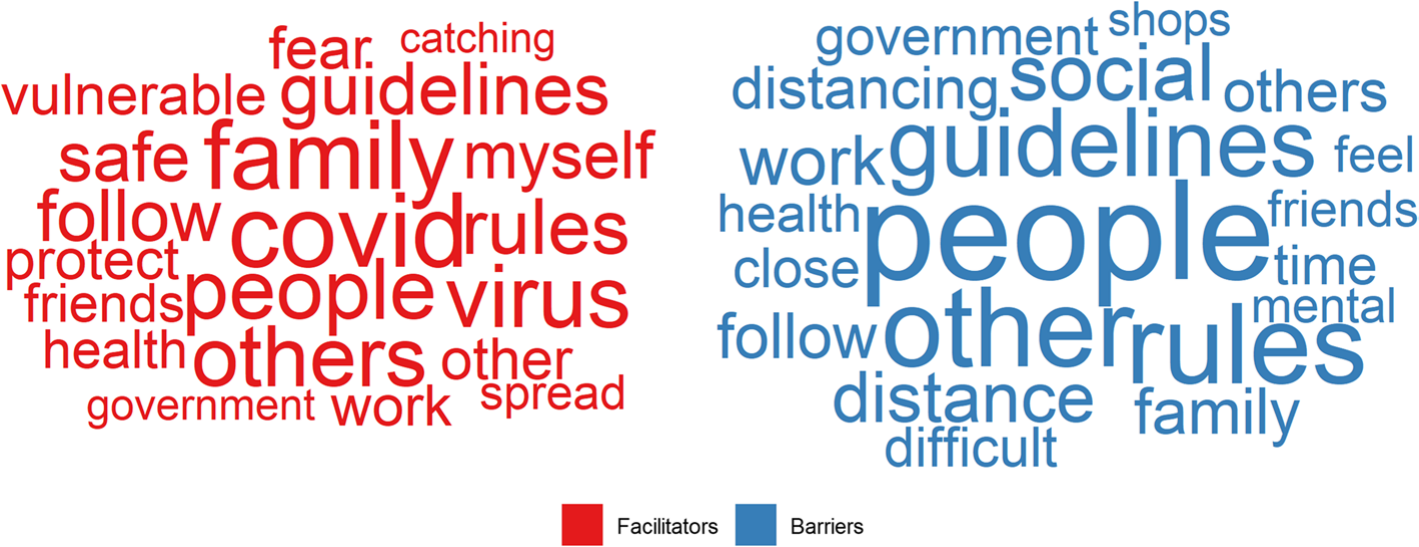
Word cloud. Twenty most frequently used words across responses, by question. Words sized according to proportion of responses they appear in

A word cloud of the twenty most frequently used words for each question is displayed in Fig. 1. For facilitators, the most used words typically related to worries about catching the virus and protecting family and friends. For barriers, the most used words related to social distancing, mental health, and family and friends.

### Facilitators of compliance

A 14-topic solution was chosen to categorise facilitators of complying with guidelines. Exemplar quotes, topic descriptions, and topic proportions are presented in Table 1. Topics are ordered according to the estimated proportion of text devoted to each topic. There was some overlap in these topics in the themes they identified and there were also some cases of topics containing multiple themes.

**Table 1:** Topic descriptions – facilitators of compliance

Topic	Proportion	Short Title	Description	FREX	Exemplar Text
F1	12.17%	Catching and transmitting COVID	Fear of catching COVID and infecting others, especially parents	fear, pass, catch, elderli, rel, dy, myself, covid, contract, longcovid	“I am terrified of being a vector for the illness and very scared of catching it myself. I particularly fear passing it on to my parents and inlaws who are all in their 70a [sic]”
F2	10.04%	Protecting high risk	Worry about catching COVID as high risk or relatives high risk	viru, underli, risk, spread, prevent, potenti, minimis, highrisk, reduc, condit	“fear of catching the virus myself or transmitting the virus eg to my 92 yo father or my partner with multiple underlying health conditions.”
F3	9.33%	Public information	Consuming media on COVID, such as government briefings	new, advic, inform, listen, scientist, tv, media, scientif, trust, watch	“The news on the T.V. The news on the Internet. Sometimes the governments bulletins. Always Listening and reading what Sir Patrick Vallance. Prof Chris Whitty. Prof Van Tam. have to say.”
F4	8.39%	Following the rules	Rule following as a personality trait. Also contains text on instrumental rule following	follow, rule, guidelin, stick, peopl, make, upset, consider, reason, adher	“I tend to just stick to rules even if I don’t necessarily agree with them” “for me, rules are rules....I may not agree with them or believe they have the outcome they suggest, but it’s the rules so I follow along”
F5	7.62%	Social responsibility	Acting out of common sense and sense of duty and civic responsibility	common, sens, respons, infect, duti, commun, civic, other, act, respect	“Common sense and a sense of responsibility to others regardless of their atitude [sic]”
F6	6.96%	Protecting vulnerable	Reducing risks for those with existing clinical vulnerabilities	vulner, extrem, clinic, protect, categori, father, husband, older, chronic, mum	“To protect myself (clinically vulnerable), my husband (same), and 3 parents (clinically extremely vulnerable).”
F7	6.62%	Reminders and accessibility	Availability of hand sanitiser and reminders to wear masks	keep, mask, wear, hand, wash, sanitis, facemask, safe, transport, sign	“Signs on shop/building entrances reminding to wear mask, provision of hand sanitiser”
F8	6.49%	Safety of loved ones	Protecting family and friends	famili, safeti, concern, healthi, ill, encourag, friend, self, safeguard, fall	“To keep my family safe. I couldn’t bear to be responsible for someone in my family falling ill with COVID-19.”
F9	6.37%	Return to normality	Following guidelines to end pandemic sooner and return society to normal.	normal, vaccin, hope, sooner, life, quickli, quicker, control, end, desir	“The hope of the pandemic ending and life being able to return to more of a normality”
F10	5.76%	Working from home / support bubbles	Ability to stay at home due to unemployment or working arrangement. Benefit of having support bubbles.	work, carehom, babi, bubbl, home, employ, ward, afford, alon, support	“Being in a support bubble as I live alone. If we did not have support bubbles I think I would have had to break the restrictions for my own mental wellbeing.” “I prefer to be at home and because I can work from home I have not had to go out much.”
F11	5.5%	Activities and Zoom	Availability of activities, including walking and going out in nature. Speaking to family, friends and online groups over Zoom.	garden, meet, walk, exercis, weather, easier, outdoor, zoom, enjoi, dog	“a structured exercise routine i.e. online Zoom classes, weekly exercise classes outside in local park and regular long cycle rides and walks”
F12	5.48%	COVID symptoms	Discussion of COVID symptoms and test results	test, april, month, march, result, surgeri, diagnos, posit, week, flu	“I was told to isolate for 6 days due to test and trace app”
F13	5.1%	Protecting the NHS	Reducing burden on NHS and its workers	nh, impact, pressur, know, worker, servic, burden, motiv, understand, frontlin	“Husband frontline NHS worker. I have heard how bad it can be for patients and staff” “Knowing that I would be helping the NHS to not become overwhelmed with patients”
F14	4.16%	Miscellaneous themes	Gallimaufry of themes, several related to those identified in Topics 1-13.	children, school, teacher, kid, young, adult, appli, grandchildren, socialis, differ	–

Several of the topics related to desires to protect oneself and others from COVID-19. The largest topic, Topic F1 (12.17%; Catching and transmitting COVID), included text on worries about catching the virus and passing it on to family members, particularly elderly parents. Topics F2 (10.04%; Protecting high risk) and Topic F6 (6.96%; Protecting vulnerable) related to protecting high risk and clinically vulnerable individuals, specifically. Exemplar texts typically referred to reducing risk for oneself and for one’s family and to a lesser extent the wider population. Similar to Topic F1, Topic F8 (6.49%; Safety of loved ones) related to protecting family and friends (topics differ in specific words used). Topic F13 (5.1%; Protecting the NHS) related to reducing the burden on the National Health Service and its workers.

Prosocial motivations were also identified in Topic F5 (7.62%; Social responsibility), which explicitly couched compliance as a matter of social responsibility, civic duty, or simply a matter of “common sense”. Topic F4 (8.39%; Following the rules) identified individuals predisposed to follow rules in general, though this topic also surfaced responses from individuals describing others’ rule following as a motivator. Topic F9 (6.37%; Return to normality) related to desires to hasten the end of the pandemic and return to life as normal.

A role of media coverage and scientific information was identified in Topic F3 (9.33%; Public information), which contained text on news and media reports, including briefings from Government ministers and scientists. Topics F7, F10 and F11 each related to factors that made compliance easier. Topic F7 (6.62%; Reminders and accessibility) included positive statements on the availability of hand sanitiser in shops and on reminders to wash hands and wear masks in public places. Topic F10 (5.76%; Working from home/support bubbles) included responses from individuals who had been able to work from home (or who had lost work and so did not need to travel to a workplace) and also on the availability of support bubbles, allowing participants to receive or give support to family and friends. Topic F11 (5.5%; Activities and Zoom) related to activities that had improved quality of life during lockdown, including walking in nature and participating in online activities, such as arts and talking to family members over Zoom.

The final two topics, Topics F12 and F14, contained irrelevant material or identified texts on a heterogeneous set of themes. Topic F12 (5.48%; COVID symptoms) included discussion of personal symptoms from COVID-19 or experiences with the test and trace system. Responses appeared to arise from participants expanding on their response to preceding questionnaire items. Topic F14 (4.16%; Miscellaneous themes) identified responses generally covering themes from the other topics, typically using slightly different phrasing.

### Compliance facilitators and respondent characteristics

**Fig. 2:**
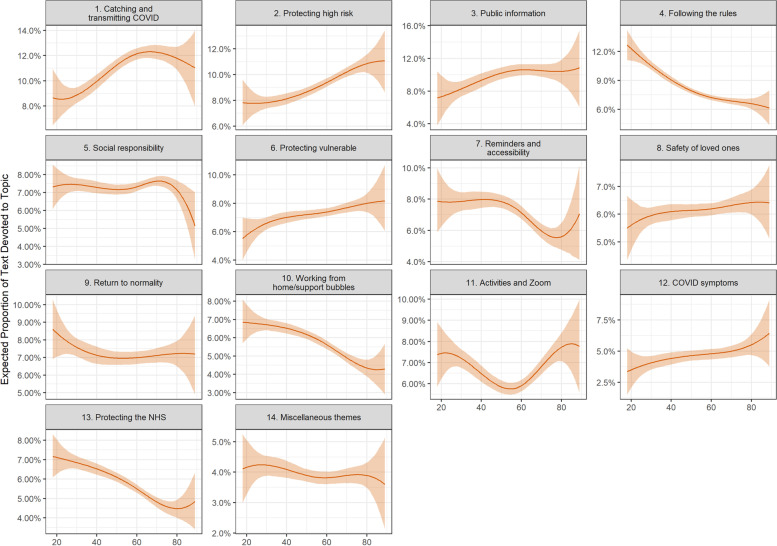
Association between facilitator document topic proportion and participant’s age (+ 95% confidence intervals). Derived from OLS regression models including adjustment for gender, ethnicity, age, education level, living arrangement, psychiatric diagnosis, long-term physical health conditions, self-isolation status, Big-5 personality traits and confidence in government

**Fig. 3:**
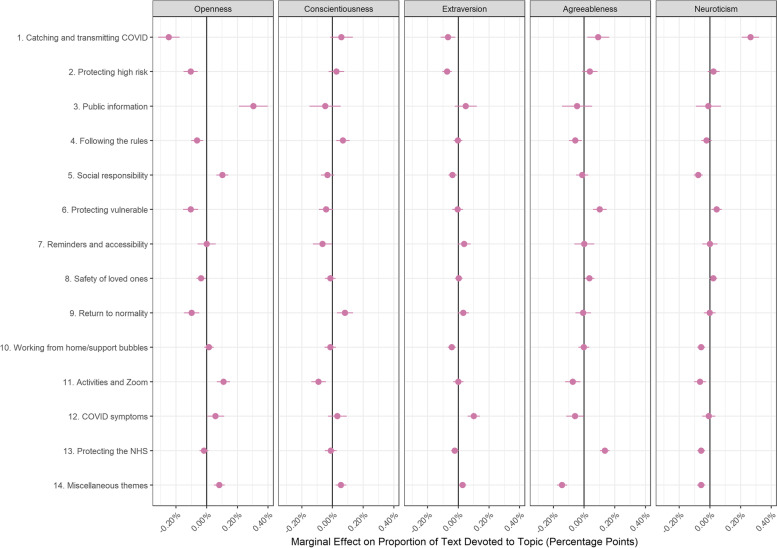
Association between facilitator document topic proportion and Big-5 personality traits (+ 95% confidence intervals). Derived from OLS regression models including adjustment for gender, ethnicity, age, education level, living arrangement, psychiatric diagnosis, long-term physical health conditions, self-isolation status, Big-5 personality traits and confidence in government

**Fig. 4:**
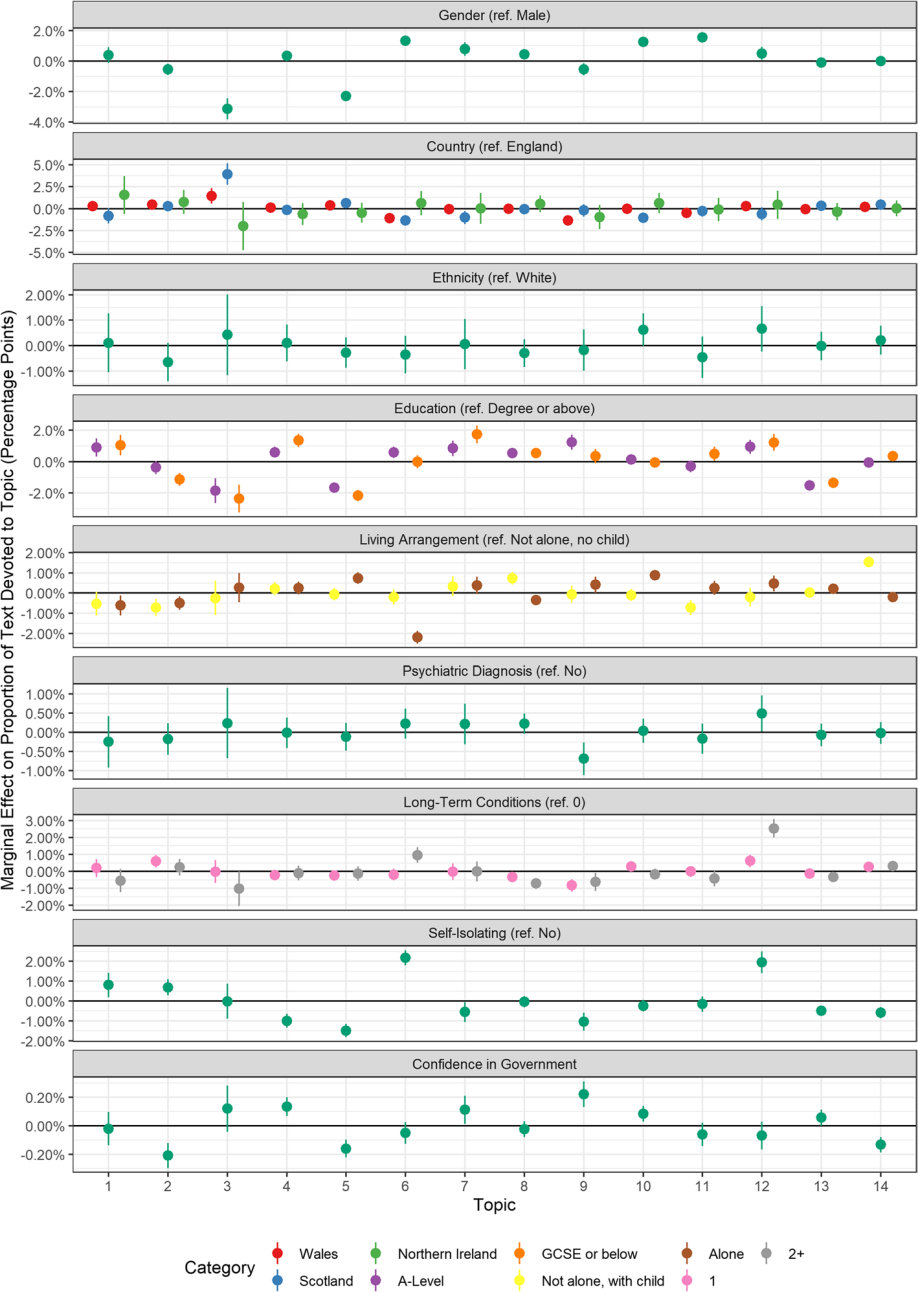
Association between facilitator document topic proportion and participants’ demographic and socioeconomic characteristics, health, and confidence in government (+ 95% confidence intervals). Derived from OLS regression models including adjustment for gender, ethnicity, age, education level, living arrangement, psychiatric diagnosis, long-term physical health conditions, self-isolation status, Big-5 personality traits and confidence in government

Figures 2, 3, 4 display the results of models regressing topic proportions on respondent characteristics. Figure 2 shows associations with age. Figure 3 shows associations with personality traits. Figure 4 shows associations with demographics, socio-economic factors, health and participants’ confidence in government. (Note, each of these results comes from models with adjustment for other measured factors.)

There were a number of differences according to age (Fig. 2). Younger participants were more likely to discuss following the rules (Topic F4), working from home/support bubble (Topic F10), and protecting the NHS (Topic F13). Older people were more likely to discuss catching and transmitting COVID (Topic F1), protecting high risk and vulnerable (Topics F2 and F6), and consuming public information (Topic F3). Interestingly, there was a U-shaped association between age and Topic F11 (activities and Zoom), with younger and older people more likely to discuss the topic than the middle aged.

There were also several differences according to personality traits (Fig. 3). Individuals high in trait openness and extraversion were less likely to discuss catching and transmitting COVID-19 (Topic F1) as a motivation to comply, while more agreeable or neurotic individuals were. Openness was also related to a higher likelihood of discussing public information (Topic F3), acting out of social responsibility (Topic F5), and finding activities and Zoom helpful (Topic F11). Conscientious individuals were more likely to discuss following the rules (Topic F4) or wanting to return to normality (Topic F9), while agreeable individuals were more likely to discuss protecting the vulnerable (Topic F6) and protecting the NHS (Topic F13). Finally, neurotic individuals were less likely to discuss social responsibility (Topic F5), working from home / support bubbles (Topic F10), activities and Zoom (Topic F11), and protecting the NHS (Topic F13), though this may partly be due to the strong association between neuroticism and discussing catching and transmitting COVID-19 (Topic F1).

Finally, there were several differences according to participants’ further demographics as well as their socio-economic characteristics, health and confidence in government (Fig. 4). Female participants were less likely to discuss Topic F3 (Public information) or Topic F5 (Social responsibility), but more likely to mention Topic F6 (Protecting vulnerable), Topic F10 (Working from home/support bubbles) or Topic F11 (Activities and Zoom). Individuals with less than degree level education were more likely to discuss Topic F4 (Following the rules) and Topic F7 (Reminders and accessibility) and less likely to discuss Topic F3 (Public information), Topic F5 (Social responsibility), or Topic F13 (protecting the NHS). Individuals who lived alone or who had not been self-isolating were less likely to mention protecting the vulnerable (Topic F6) and individuals with psychiatric diagnoses were more likely to discuss returning to normality as a motivator (Topic F9). Individuals with greater confidence in government were more likely to discuss following the rules (Topic F4) and wanting to return to normality (Topic F9). They were also less likely to mention protecting high risk (Topic F2) or acting out of social responsibility (Topic F5).

### Barriers to compliance

We selected a 14-topic solution for the responses on barriers to compliance. Short descriptions are displayed in Table 2, along with exemplar quotes and topic titles that we use when plotting results. Three topics related to difficulties complying due to the actions of other people. The largest topic (Topic B1; 20.82%; Others invading space) identified responses on difficulties social distancing in public places (i.e., remaining 2 m apart) due to others getting too close, particularly in supermarkets or on pavements. Topic B8 (6.43%; Workplace issues) related to issues in workplaces that prevented people from being able to comply. The topic surfaced several responses from teachers describing the challenge of maintaining social distancing with hundreds of schoolchildren. Topic B10 (5.74%; Social norms and social pressures) related to challenges resisting social pressure to break rules from family and friends, and well as the specific demotivation of seeing non-compliance among the general public and members of the government.

**Table 2:** Topic descriptions – barriers to compliance

Topic	Proportion	Short Title	Description	FREX	Exemplar Text
B1	20.82%	Others invading space	Difficulty of maintaining social distancing in public, due to actions of others, particularly in supermarkets.	space, supermarket, distanc, maintain, social, difficult, other, observ, street, crowd	“Whilst food shopping I keep my distance from others.. however many people do not keep their distance from me. Unbelievable how many people reach over /past me to reach something from shelves i am stood in front of”
B2	9.79%	Issues with masks and sanitiser	Forgetting to wear masks, wash hands, or use hand sanitiser. Discomfort caused by masks or santiser.	mask, wear, hand, forget, wash, sanitis, deliveri, breath, facemask, dog	“Shortness of breath and asthma means that I don’t wear a face covering but I do wear an exemption badge.” “Forgetfulness: workmen renovating my flat nearby, occasionally have forgotten to wear a mask or wash/gel hands - re latter often remember some time after but worry it is too late”
B3	7.91%	Mental health and family support	Impacts on own and family members mental health and insufficiency of support bubbles to meet practical needs.	mental, health, support, mother, bubbl, elderli, childcar, relationship, dad, carer	“Single parent with twin toddlers, need support from more than one bubble for childcare and to support mental health,” “Mental health issues for myself and relatives. My daughter struggles with not being able to see her boyfriend.”
B4	7.24%	Special circumstances	Breaking the rules on occasion, usually due to necessity or emotional wellbeing	hous, week, grandchildren, 3, birthdai, grandson, old, 6, hug, 7	“I look after my grandchildren so kissing and hugging happens, my daughters had their birthdays so we visited to give presents and hugged them, terrible to feel guilty hugging your children. My 85 year old father asks for shopping occasionally, we take it to him and enter the house but feel guilty doing so.” “I look after my grandson 1 day a week & babysit at other times. I have 2 adult sons who moved out recently & come back to the house to get things.”
B5	7.07%	Following the guidelines	Following the guidelines completely to the best of one’s knowledge and ability.	guidelin, follow, obstacl, transport, line, public, easi, selfish, guid, complet	“I have tried to follow guide lines 100% but have probably slipped up a few times” “I always follow the guidelines.”
B6	6.56%	Missing family and friends	Desire to meet family and friends. Meeting outside made harder due to poor weather.	friend, miss, garden, famili, christma, allow, meet, spend, coffe, socialis	“Missing family. In summer the weather made it easier and we could meet in the garden, this isn’t so tempting in winter!”
B7	6.55%	Lack of trust in government	Lack of trust and confidence in government competence. Anger at Dominic Cummings.	advic, messag, cum, trust, domin, minist, bori, faith, incompet, johnson	“Boris Johnson, lack of leadership, lack of decisive and timely advice, mixed messages, Dominic Cummings idiocy, continuing lies from Ministers” “lack of confidence in the evidence base for decision-making, lack of confidence that decision-makers are acting in best interests of population”
B8	6.43%	Workplace issues	Difficulty complying with rules in the workplace, especially in schools.	school, teacher, work, colleagu, primari, student, offic, teach, workplac, meter	“The rules being had to implement in a work environment (secondary school) it’s not possible to remain 1 m away from pupils in corridors or in classrooms when there’s 30+ in the class” “I am considered an essential worker and have to go to work. Although some of us could work from home we all have to attend the office every day and mix with colleagues.”
B9	6.39%	Perceiving the risks as low	Belief that the risk is low or that the statistics are inaccurate or exaggerated. Also contains text on difficulties with testing or the Test and Trace system.	covid, test, low, catch, viru, risk, life, symptom, posit, normal	“I am in an area with very low infection rates and still don’t think I know anyone who has actually tested positive or been very ill.” “I don’t believe the stats in the media and the risks associated with COVID-19 have been completely over exaggerated and are killing the economy. Deaths in hospital are falsely being recorded as COVID-19 and the percentages of people who have died are tiny and do not warrant lockdowns. The few people I know who have tested positive, have not even known they had it. I have zero trust in any facts presented about COVID-19 and therefore I am not going to stop seeing my loved ones.”
B10	5.74%	Social norms and social pressures	Pressure from family, friends and colleagues to bend rules. Observation of others – including those in power – breaking rules. Also, difficulties related to confusion around the rules.	pressur, stick, break, relax, make, peer, habit, guidanc, rule, tempt	“I have been following the guidelines but I am always made to feel like grinch by colleagues who bend rules to suit themselves. Its (sic) hard” “I have been following guidelines, but I have seen so many people not following the guidelines it makes me more tempted to ‘bend the rules’ slightly.”
B11	4.35%	Complexity of rules	Difficulties following frequently changing rules that are also perceived as lacking logical basis. Lack of flexibility in rules.	sens, chang, tier, common, logic, complic, stupid, ridicul, nonsens, appli	“Stupid nonsensical non-evidenced government knee jerk reactions eg closing places of worship and golf clubs with no real rationale.” “I think we should be using common sense rather than overly prescriptive rules that don’t always make sense”
B12	4.16%	Loneliness	Impact on mental wellbeing of living alone.	live, alon, human, interact, limit, loneli, person, contact, period, lowrisk	“I need face to face human contact for my mental well being and my sanity” “I live on my own, and so do the majority of people I interact with. The restriction to meet as two households outside means I can really only ever see one other person, and that really limits meaningful shared experiences which I think are important for my mental health. I have on occasion broken this rule to meet e.g. as three of us outside.”
B13	3.54%	Geographical variation in rules	Difficulties related to differences in rules across areas, particularly for those living on the border between England and Wales.	england, wale, border, understand, letter, attitud, action, measur, interpret, consist	“I live right on the border between Wales and England and have not been able to stay entirely within Wales at times when the border was closed because of the locations of essential local services” “Frequency of changes to guidelines. Differences between devolved and English measures. English measures have been more clearly communicated. I’m still uncertain on Welsh rules for meeting other people.”
B14	3.45%	Confusion around rules	Confusion about what the rules are. Also contains text on adhering with guidelines to best of one’s ability.	hinder, frustrat, applic, regul, awar, adher, situat, confus, abil, disregard	“The instructions are confused and confusing. The variety of scientific opinion is not always reflected but interviewers seem sometimes more interested in apportioning blame than establishing consensus.”

Several topics related to difficulty understanding current rules. Topic B11 (4.35%; Complexity of rules) identified individuals who had difficulty keeping abreast of (frequently changing) rules or who could not understand the logic behind the rules (for instance, keeping pubs open but stipulating only four people could meet). Some responses identified in this topic also questioned the lack of flexibility in rules or the allowance for individuals to apply common sense. Topic B13 (3.54%; Geographical variation in rules) related to challenges arising from the variation in rules around the UK, particularly for those living near borders, such as that between England and Wales. One frequently noted issue was accessing essential services located on the other side of a border. Topic B14 (3.45%; Confusion around rules) included responses that described general confusion about what the rules were, though this topic also identified individuals who stated following guidelines to the best of their ability. (Topic B5 [7.07%; Following the guidelines] also related to individuals stating that they had been following guidelines completely.) Criticism of the government was voiced in Topic B7 (6.55%; Lack of trust in government), with individuals noting a lack of confidence or trust in the government’s decisions or motives, including in Boris Johnson’s leadership. Several participants also expressed anger at Dominic Cummings – a senior Government advisor – and the decision to keep him in post following reports in May 2020 that he had broken lockdown rules.

Several topics related to the negative impact of compliance on the lives of participants and their family and friends. Topic B3 (7.91%; Mental health and family support) related directly to the impact of restrictions on mental health for the participant themselves and their family and friends. The topic also identified responses from individuals who had extended support bubbles beyond the rules to provide or receive support from loved ones. This included both emotional and practical support (e.g., by providing childcare). Topic B6 (6.56%; Missing family and friends) related to difficulties due to missing family and friends, particularly when the weather turned poor and meeting outside became a challenge. Topic B12 (4.16%; Loneliness) identified responses from individuals who lived alone and had struggled due to the resulting social isolation. Some individuals described breaking lockdown rules on occasion for human contact. Topic B4 (7.24%; Special circumstances) also identified individuals who had occasionally broken lockdown rules to improve wellbeing, but also identified individuals breaking rules to provide support to family members (e.g., childcare) or out of temporary necessity (e.g., helping a family member move out of their home).

Practical barriers to compliance were reflected in Topic B2 (9.79%; Issues with masks and sanitiser) which related to difficulties with face masks or using hand sanitiser. Several respondents stated forgetting to wear masks or use sanitiser or noted discomfort from the use of these due to existing health conditions. Finally, Topic B9 (6.39%; Perceiving the risks as low) identified individuals who did not comply with guidelines due to perceptions that risks were low, for instance, due to previous infection with COVID, low caseloads in the local area, or beliefs that government statistics were exaggerated. The topic also identified a number of responses of individuals who had not gotten tested when displaying symptoms, either due to lack of availability or beliefs that PCR tests were not accurate.

### Compliance barriers and respondent characteristics

**Fig. 5:**
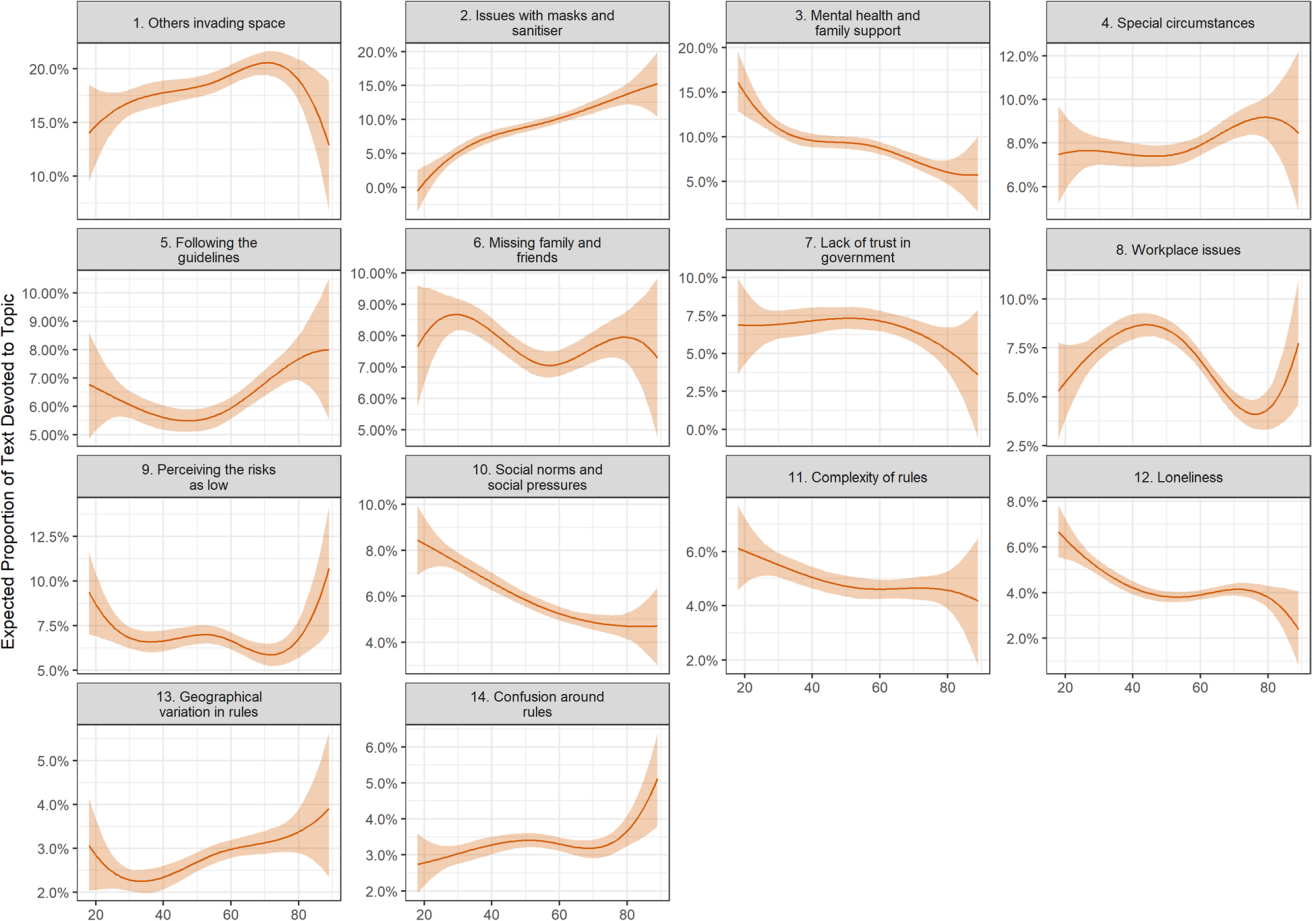
Association between barrier document topic proportion and participant’s age (+ 95% confidence intervals). Derived from OLS regression models including adjustment for gender, ethnicity, age, education level, living arrangement, psychiatric diagnosis, long-term physical health conditions, self-isolation status, Big-5 personality traits and confidence in government

**Fig. 6:**
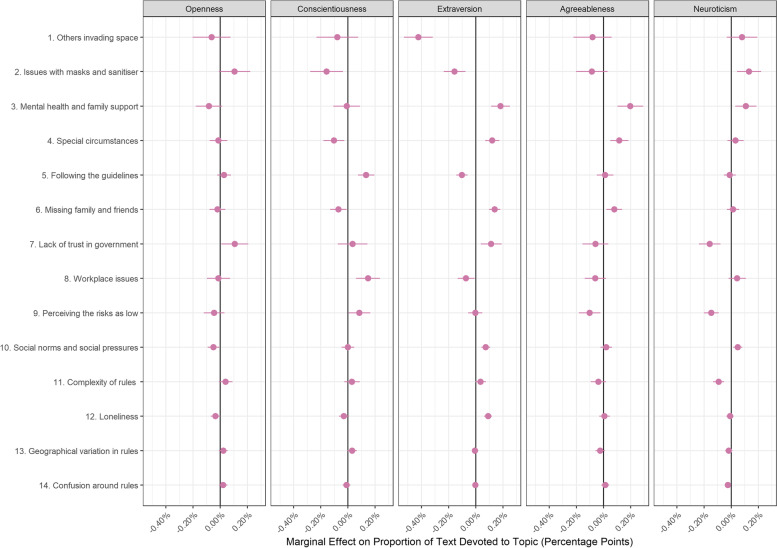
Association between barrier document topic proportion and Big-5 personality traits (+ 95% confidence intervals). Derived from OLS regression models including adjustment for gender, ethnicity, age, education level, living arrangement, psychiatric diagnosis, long-term physical health conditions, self-isolation status, Big-5 personality traits and confidence in government

**Fig. 7:**
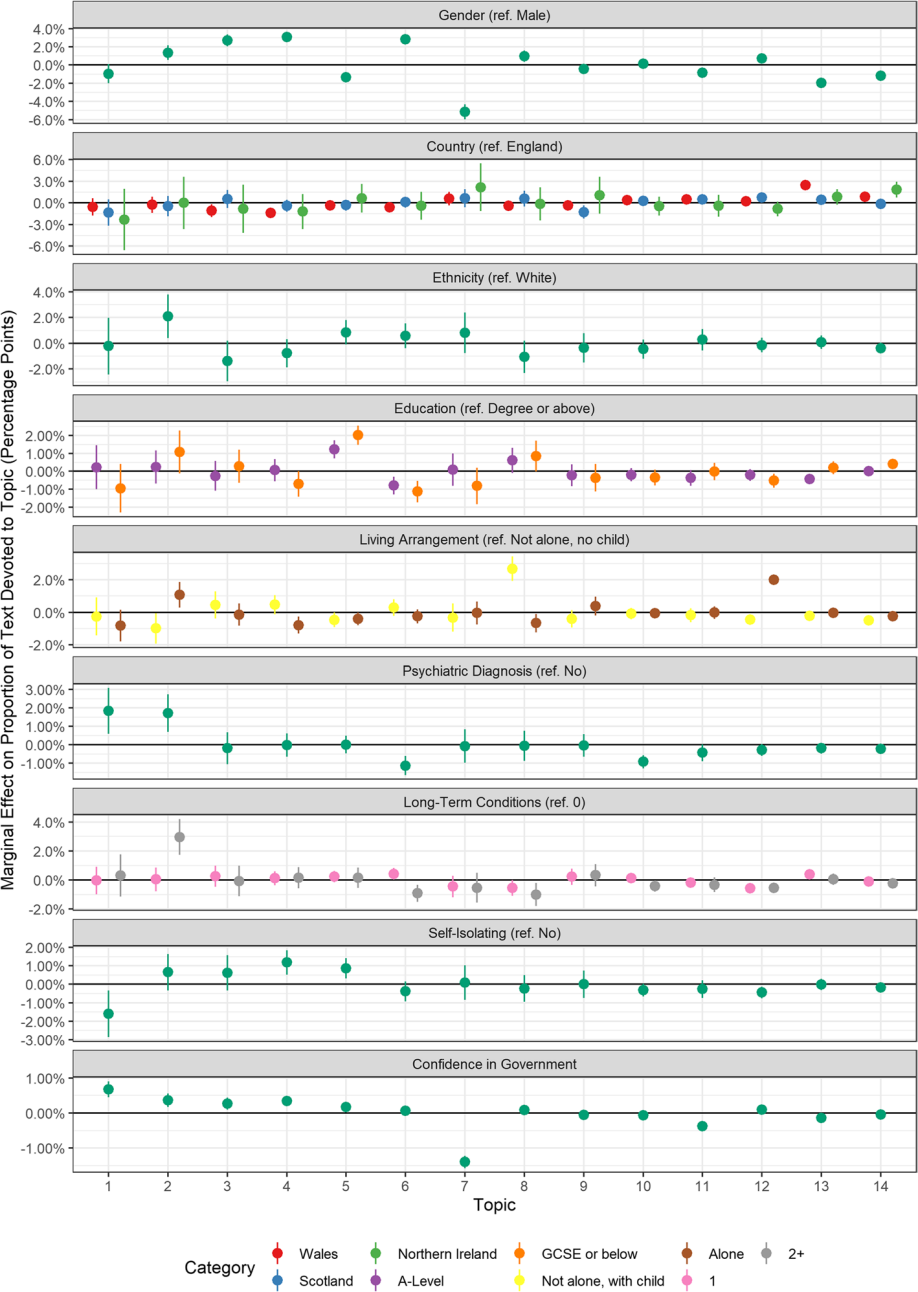
Association between barrier document topic proportion and participants’ demographic and socioeconomic characteristics, health, and confidence in government (+ 95% confidence intervals). Derived from OLS regression models including adjustment for gender, ethnicity, age, education level, living arrangement, psychiatric diagnosis, long-term physical health conditions, self-isolation status, Big-5 personality traits and confidence in government

Figures 5, 6 and 7 display the results of models regressing topic proportions on respondent characteristics. There were a number of differences according to age (Fig. 5). Older participants were more likely to discuss issues with masks and sanitiser (Topic B2) and geographic variation in rules (Topic B13) and confusion around rules (Topic B14). Young people were more likely to mention mental health and family support (Topic B3), social pressures (Topic B10), and loneliness and social isolation (Topic B12).

There were also a number of differences according to personality traits (Fig. 6). Notably, extraverted individuals were less likely to discuss others invading space (Topic B1), having issues with masks or sanitiser (Topic B2) or following the guidelines completely (Topic B5) but were more likely to mention social factors such as mental health and family support (Topic B3), breaking guidelines on occasion (Topic B4), missing family and friends (Topic B6), difficulties with social norms and social pressures (Topic B10), and loneliness (Topic B12). Neurotic individuals were less likely to discuss COVID as being low risk, and conscientious individuals were more likely to discuss following the guidelines completely (Topic B5).

Finally, there were several differences according to participants’ demographic and socio-economic characteristics, health and confidence in government (Fig. 7). Female participants were more likely to discuss Topic B3 (Mental health and family support), Topic B4 (Special circumstances), and Topic B5 (Following the guidelines) and less likely to discuss lack of trust in government (Topic B7). Individuals with less than degree level education were also more likely to state they were following the guidelines completely (Topic B5) but were less likely to discuss missing family and friends (Topic B6). Individuals with children were more likely to discuss workplace issues (Topic B8), while individuals with psychiatric diagnoses were more likely to mention others invading space (Topic B1) or having issues with masks and sanitiser (Topic B2). Supporting our structural topic models, individuals with high confidence in government were less likely to discuss Topic B7 (lack of trust in government) and individuals who lived alone were more likely to discuss loneliness (Topic B12).

### Associations between topic proportions and self-reported compliance

**Fig. 8:**
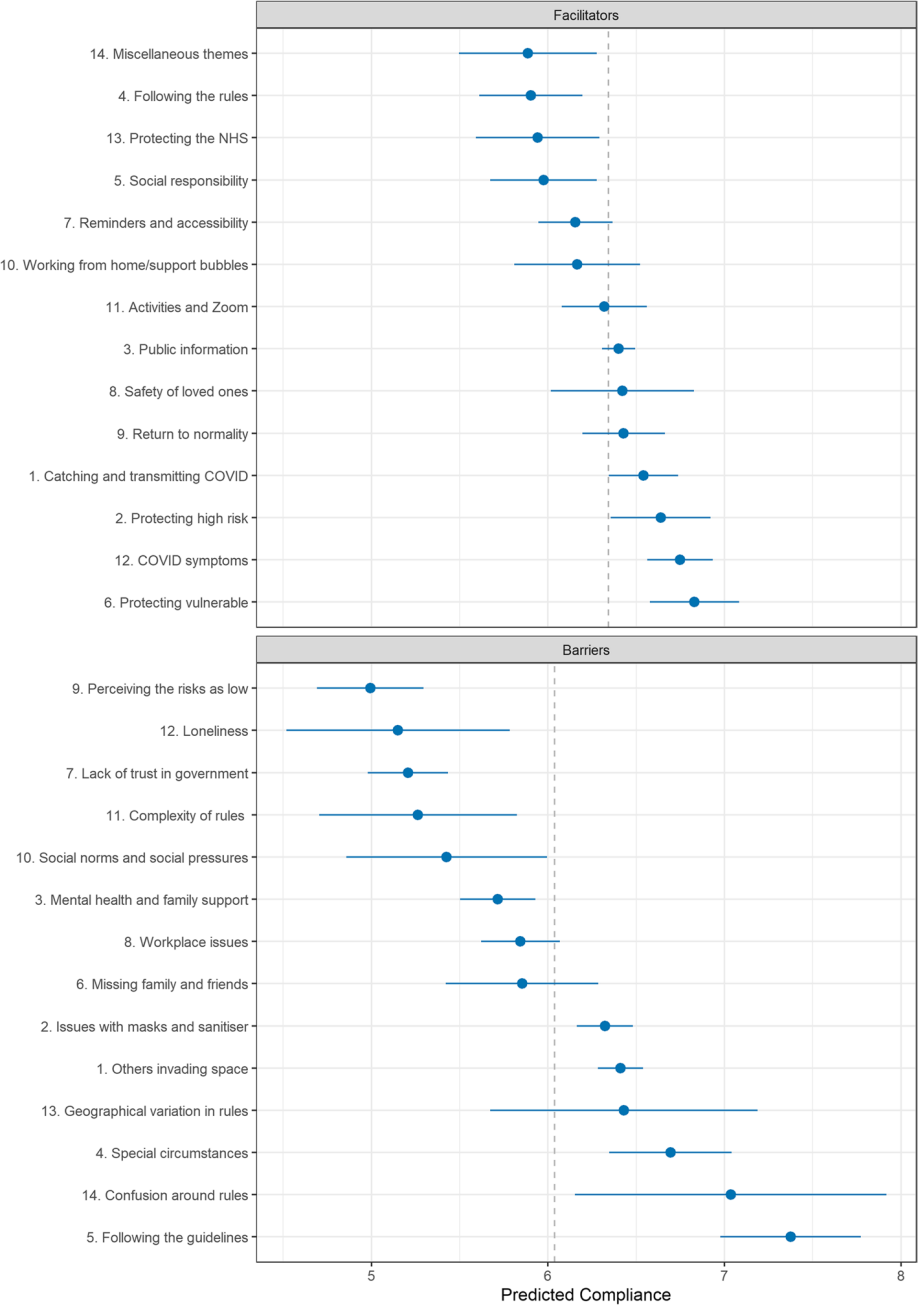
Association between self-reported compliance with COVID-19 related guidelines and document topic proportions (+ 95% confidence intervals). Dashed line indicates means compliance levels in sample used in regression

The results of regressions estimating the average level of self-reported compliance according to topic proportions are displayed in Fig. 8. (The dashed line represents the mean compliance level across the relevant sample.) The top panel shows results for facilitators and the bottom shows results for barriers to compliance.

Facilitator topics related to desires to protect the vulnerable (Topic F6) or high risk (Topic F2) or reduce the likelihood of catching and transmitting COVID-19 were associated with highest compliance levels. Following the rules (Topic F4), desire to protect the NHS (Topic F13) and acting out of social responsibility (Topic F5) were related to lowest average compliance levels. For barriers to compliance, perceiving COVID-19 as representing a low risk (Topic B9), living alone (Topic B12), lack of trust in government (Topic B7) and finding the rules to be too complex (Topic B11) was related to lowest compliance levels. Following the guidelines completely (Topic B5), confusion around the rules (Topic B14) and facing special circumstances (Topic B4) were related to higher than average compliance.

## Discussion

Using free-text data from over 17,500 adults, we identified several facilitators and barriers to compliance with COVID-19 guidelines, 8 months after lockdown measures were implemented in the UK. For facilitators of compliance, a sizeable proportion of text was related to desire to reduce risks for oneself, one’s family and friends, and – to a lesser extent – the general public and the NHS and its workers, specifically. Some participants also spoke of being motivated by a sense of responsibility, a desire to return to life as normal, or acting from a predisposition to follow rules in general. For barriers to compliance, a substantial proportion of text was related to other people making compliance difficult, either by getting too close in public or workplaces, putting social pressure on participants to violate guidelines, or acting as a demotivator when their non-compliance was observed by participants. Participants also spoke of the emotional toll of complying with guidelines, particularly among those who lived alone, missing family and friends, and of the necessity of providing support to loved ones. Interestingly, participants also spoke of breaking rules on occasion, for instance when social isolation had gotten too great. A large portion of text was also devoted to issues with the guidelines themselves. Participants found guidelines confusing and often did not see their logical basis. The variation in rules across time and geographic areas was a particular issue. Some participants also discussed a lack of trust in government and expressed anger at the decision to keep Dominic Cummings in his position as government advisor after he broke lockdown rules. Last, some participants discussed believing COVID-19 to be low risk as a reason for not complying. Individuals who spoke about this had the lowest self-reported compliance overall.

Several theoretical frameworks have been used to understand individual differences in compliance with preventive behaviours. One fruitful framework has been the COM-B model [16, 35]. The COM-B model [36, 37] posits that behaviour results from the interaction of physical and psychological attributes of the individual (Capability), autonomic and reflexive mental processes directing and energising behaviour (Motivation), and physical and social attributes of the environment (Opportunity). For instance, a person may enact a specific preventive behaviour (e.g., social distancing) if they have the knowledge that the behaviour is effective (psychological capability), are worried about catching COVID-19 (reflexive motivation), and do not perceive a strong social norm to act otherwise (social opportunity).

In Table 3, we map the topics identified in this analysis to components of the COM-B framework. Most of the enablers to comply were related to reflective motivation that complying would reduce adverse events and support in the return to normality. People also reported that compliance was enabled when there were clear physical opportunities to comply. Barriers to compliance were also related to reflective motivations but also to psychological capabilities and lack of social opportunity. This suggests that behaviour change techniques such as education (e.g. increasing understanding about the virus), persuasion (e.g. stimulating action through inducing positive emotions around the benefits of compliance to society), and incentivisation (e.g. communicating how better compliance could lead to lower virus levels and less need for strict measures) could help to improve motivation, whilst clearer rules, identification and removal of factors hindering compliance, and restructuring of environments (such as shops) to facilitate compliance could help to address the barriers identified [36].

**Table 3:** Mapping of topics onto COM-B framework

Topic		Capability		Motivation		Opportunity	
		Physical	Psychological	Reflective	Autonomic	Physical	Social
F1	Catching and transmitting COVID			X			
F2	Protecting high risk			X			X
F3	Public information		X	X			
F4	Following the rules				X		
F5	Social responsibility			X	X		
F6	Protecting vulnerable			X			X
F7	Reminders and accessibility				X	X	
F8	Safety of loved ones			X	X		
F9	Return to normality			X			
F10	Working from home / support bubbles					X	
F11	Activities and Zoom		X			X	X
F12	COVID symptoms			X		X	
F13	Protecting the NHS			X			
F14	Miscellaneous themes						
B1	Others invading space					X	
B2	Issues with masks and sanitiser	X					
B3	Mental health and family support		X				X
B4	Special circumstances				X		X
B5	Following the guidelines			X	X		
B6	Missing family and friends			X		X	X
B7	Lack of trust in government			X			
B8	Workplace issues					X	
B9	Perceiving the risks as low			X			
B10	Social norms and social pressures						X
B11	Complexity of rules		X				
B12	Loneliness			X			
B13	Geographical variation in rules		X				
B14	Confusion around rules		X				

The topics that participants discussed were related to participant characteristics. Notably, younger individuals were more likely to mention social pressures as a barrier to compliance, and older individuals were more likely to discuss confusion around rules. Extraverted individuals and women were more likely to report emotional challenges and lack of social contact as a barrier to compliance, while conscientious individuals were more likely to state complying with guidelines completely. The topics discussed were also likely to have differed by the date the free-text data were collected, though this was not directly tested in the present study. In particular, individuals may have been less likely to discuss protecting the vulnerable when COVID-19 cases were low in early November, and may have expressed more difficulty with isolation as Christmas approached following Government announcements that household mixing would be limited. The date on which data were collected is also likely to have influenced associations with our measure of self-reported compliance, given that the latter item regarded present compliance. In other work, we have shown self-reported full compliance tracked caseloads whilst become slightly less common over time [5].

Our results are consistent with those of Coroiu et al. [16], who study the barriers and facilitators of compliance with COVID-19 social distancing and shelter-in-place rules. The authors found that a desire to protect oneself and others and acting out of a sense of social responsibility were among the most endorsed items on motivations to comply. They also found that, among the barriers to compliance, believing the risk of catching COVID-19 to be small, not trusting government messaging, needing to provide support to friends or family, and feeling stressed when socially isolating were the most endorsed factors. We add to their results by finding that protecting family or friends appears to be a more important motivation than protecting wider society and that confusion about specific rules is a major barrier to compliance.

Our finding that a number of participants violated guidelines on occasion for emotional reasons is important in light of debates on behavioural fatigue [4, 9, 10, 12]. Existing tests of behavioural fatigue have assumed that fatigue would lead to decreasing motivation to comply across the pandemic [4, 5], a test that is in line with what England’s Chief Medical Officer, Chris Whitty, seemingly had in mind when discussing the concept (“Once we have started these things we have to continue them through the peak, and there is a risk that, if we go too early, people will understandably get fatigued and it will be difficult to sustain this over time.” [8]). Our results indicate that some individuals violate rules after a period of sustained compliance, but return to complying, a process akin to resting after a workout [9]. The implication of this may be that individuals can comply over extended periods, if occasional opportunities to “reset” are available.

Our finding that many participants had struggled to understand or keep abreast of changing rules (“alert fatigue”) was consistent with previous qualitative work showing difficulties understanding government messaging [25, 26, 38]. The results suggest that simplified rules may improve compliance, though this would have to be balanced against the cost of keeping some individuals under strict measures for longer than necessary. The lack of a clear rationale for certain rules was cited as reducing motivation to comply suggests that the UK government could increase the transparency of its decision making and that this may improve the ability to tackle COVID-19. Our finding that low confidence in government was a barrier to compliance was consistent with several previous quantitative studies [22–24] and is concerning in light of the decrease in confidence in the UK government that occurred from the beginning of the pandemic to the time of the study [5, 39], but it also offers hope for future compliance given the increase in confidence following the rollout of the vaccine [39].

Also of policy interest was the role of mental health and the need to receive or provide support to family and friends as a barrier to compliance. Social bubbles were not introduced immediately by the governments of UK. The results here suggest this rule may have caused stress or been ignored by some households. Further, some participants complained that support bubbles were of insufficient size when introduced. More flexibility may have improved the wellbeing of individuals requiring support, though an issue is that flexibility could increase the complexity of rules, which as discussed, can raise its own issues for facilitating high compliance.

This study had several strengths. We used rich qualitative data from over 17,500 UK adults representing a wide range of demographic groups. Using structural topic modelling, we were able to combine qualitative and quantitative approaches, to identify unique facilitators and barriers to compliance with guidelines in the UK, and to assess how response topics differed according to participant characteristics. Our results have several policy implications and showed (to our knowledge) unique evidence of occasional, isolated rule violations among some of the general public. The results also add detail to previous quantitative results showing a link between age, personality traits and compliance behaviour [5, 14, 21]. The likelihood of discussing some topics was associated with participant characteristics in the expected direction – for instance, individuals with low confidence in government were more likely to offer criticisms of the government – which suggests that the structural topic models extracted consistent and meaningful themes. Further, we used data from 8 to 9 months after the first lockdown, later than much of the literature on compliance during COVID-19, which has focused on the early stages of the pandemic.

Nevertheless, this study had several limitations. Not all of the topics identified a single theme consistently. Associations with participant characteristics could be driven or biased by idiosyncratic texts. We used a convenience sample that, though heterogeneous, was unrepresentative of the UK population and, further, respondents to the free-text question were biased towards the highly educated. Response biases could also have generated bias in the topic regression results. The sample was drawn from an ongoing study with frequent follow-ups (weekly from March to August 2020 then monthly thereafter). Participation in a study focused on COVID-19 could feasibly have influenced individuals’ compliance behaviour. As we focused on topics offered spontaneously by respondents, a participant not writing about a particular topic does not necessarily imply that the participant does not agree with its sentiment, though our assumptions is that the proportion of text devoted to a topic is related to its perceived importance. Finally, while we included a wide set of predictors in our structural topic models, many relevant factors were unobserved and several were likely to be measured with error or insufficient granularity. For instance, we measured ethnicity as white or non-white and geographic variation at the country level, while there is more heterogeneity within these groups. Associations may therefore be biased by unobserved confounding.

## Conclusions

We identified several facilitators and barriers to compliance. Of particular importance for facilitating compliance was concerns about the risk of COVID-19 for oneself and one’s family and friends, while important barriers to compliance were problems maintaining social distance in public spaces and difficulties understanding and keeping abreast of government rules. The results suggest that government communication that emphasises the potential risks of COVID-19 and provides simple, consistent guidance on how to reduce the spread of the virus would improve compliance with preventive behaviours. Investments in managing or reorganising public space – particularly in supermarkets – so that social distancing is encouraged could also have significant positive effects. While a large literature has related individual characteristics to preventive behaviour, our results give fresh insight into the wider contextual issues that are important for compliance.

## Supplementary Information

**Additional file 1.**

## Data Availability

The datasets generated and/or analysed during the current study are not publicly available due stipulations set out by the ethics committee but are available from the corresponding author on reasonable request. The code used to run the analysis is available at https://osf.io/nf4m9/.
