# Correction to: Guidelines: a structural topic modelling analysis of free-text data from 17,500 UK adults

**DOI:** 10.1186/s12889-022-12614-1

**Published:** 2022-03-24

**Authors:** Liam Wright, Elise Paul, Andrew Steptoe, Daisy Fancourt

**Affiliations:** 1 Institute of Education, University College London, 55-59 Gordon Square, London, WC1H 0NU UK; 2 Department of Behavioural Science and Health, University College London, 1-19 Torrington Place, London, WC1E 7HB UK

**Correction to: BMC Public Health 22, 34 (2022)**

**https://doi.org/10.1186/s12889-021-12372-6**

Following publication of the original article [1], the authors identified an error in one of the variables in their code. This has caused:

- An incorrect Figure 2
- An incorrect Figure 5
- Several textual errors in the following sections:
  - ° Compliance facilitators and respondent characteristics
  - ° Compliance Barriers and Respondent Characteristics
  - ° Discussion

These errors do not impact the conclusions/outcome of the article.

The incorrect and correct figures are shown in this correction article as figs 1-4. The text edits are available in supplementary file 1. The original article has been updated with the correct information.

**Incorrect figure 2 as originally published** 
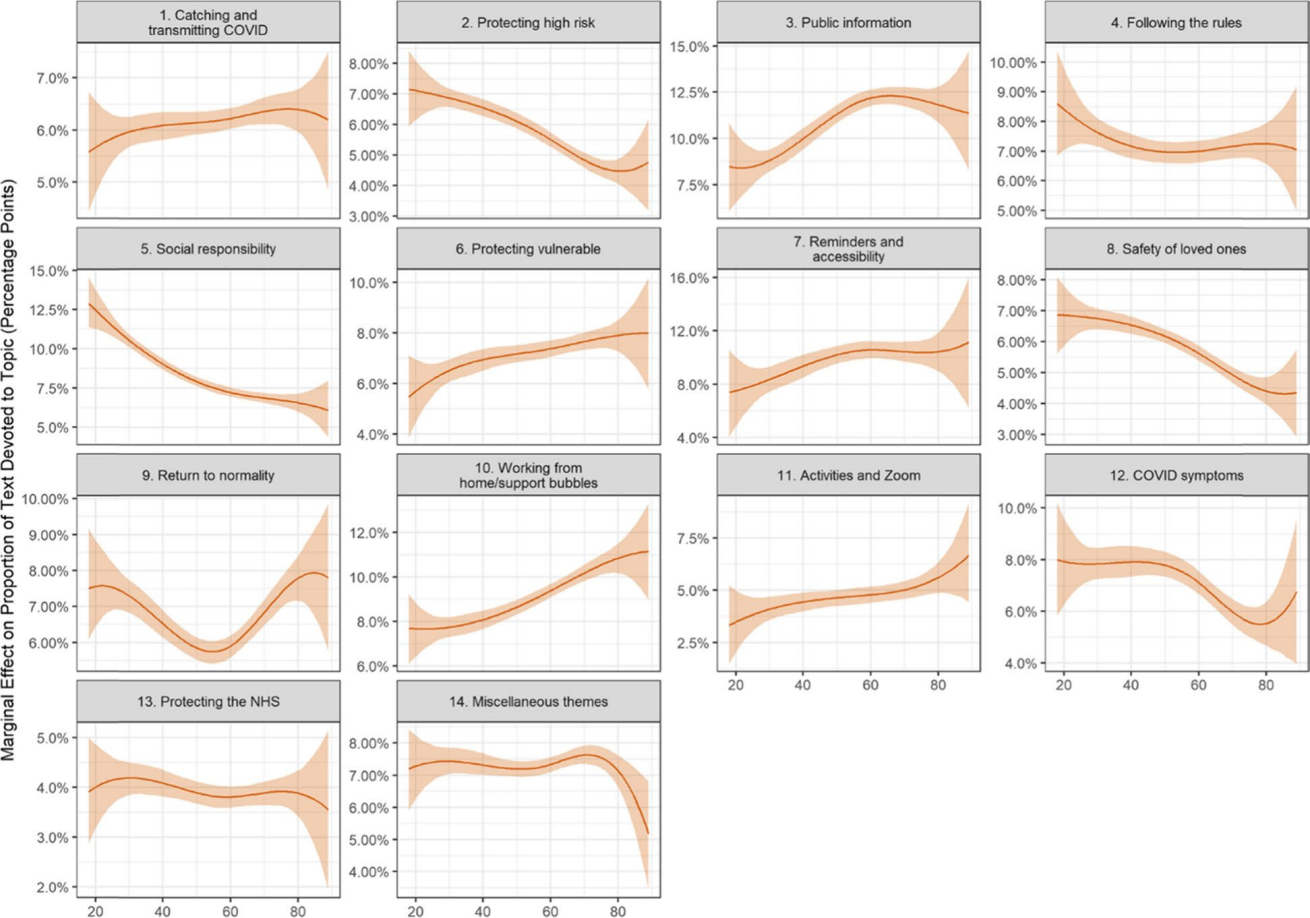


**Figure 2 Correct figure 2** 
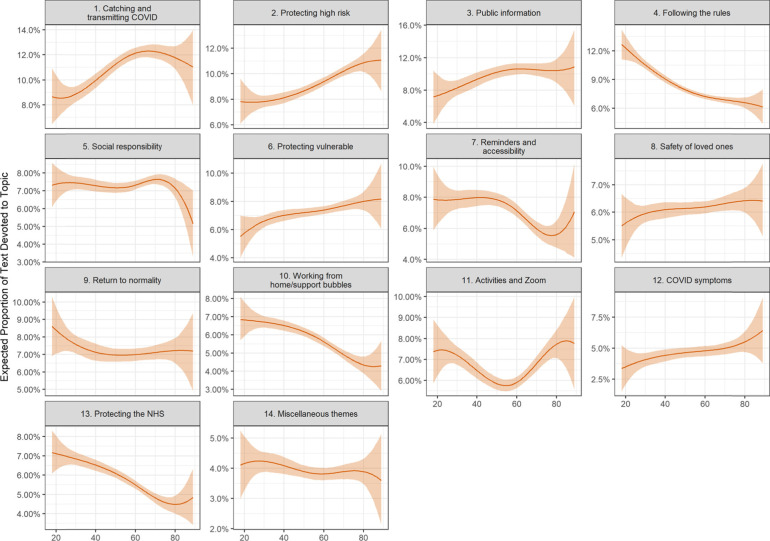


**Incorrect figure 5 as originally published** 
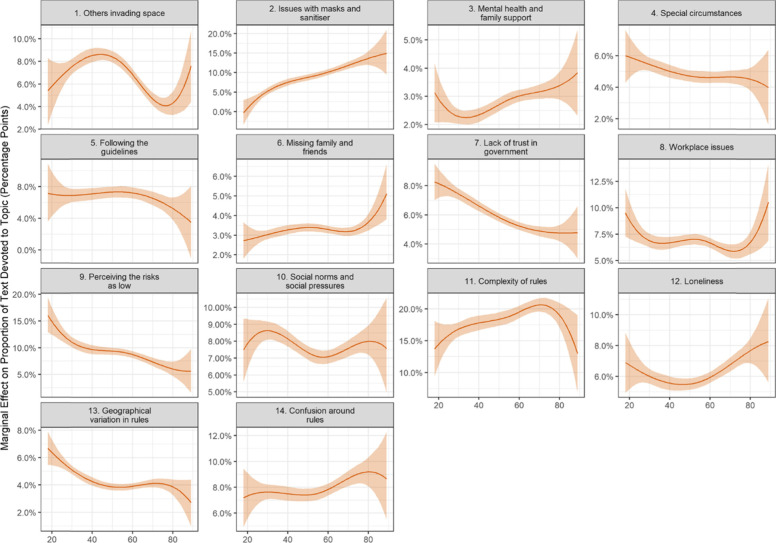


**Figure 4 Correct figure 5** 
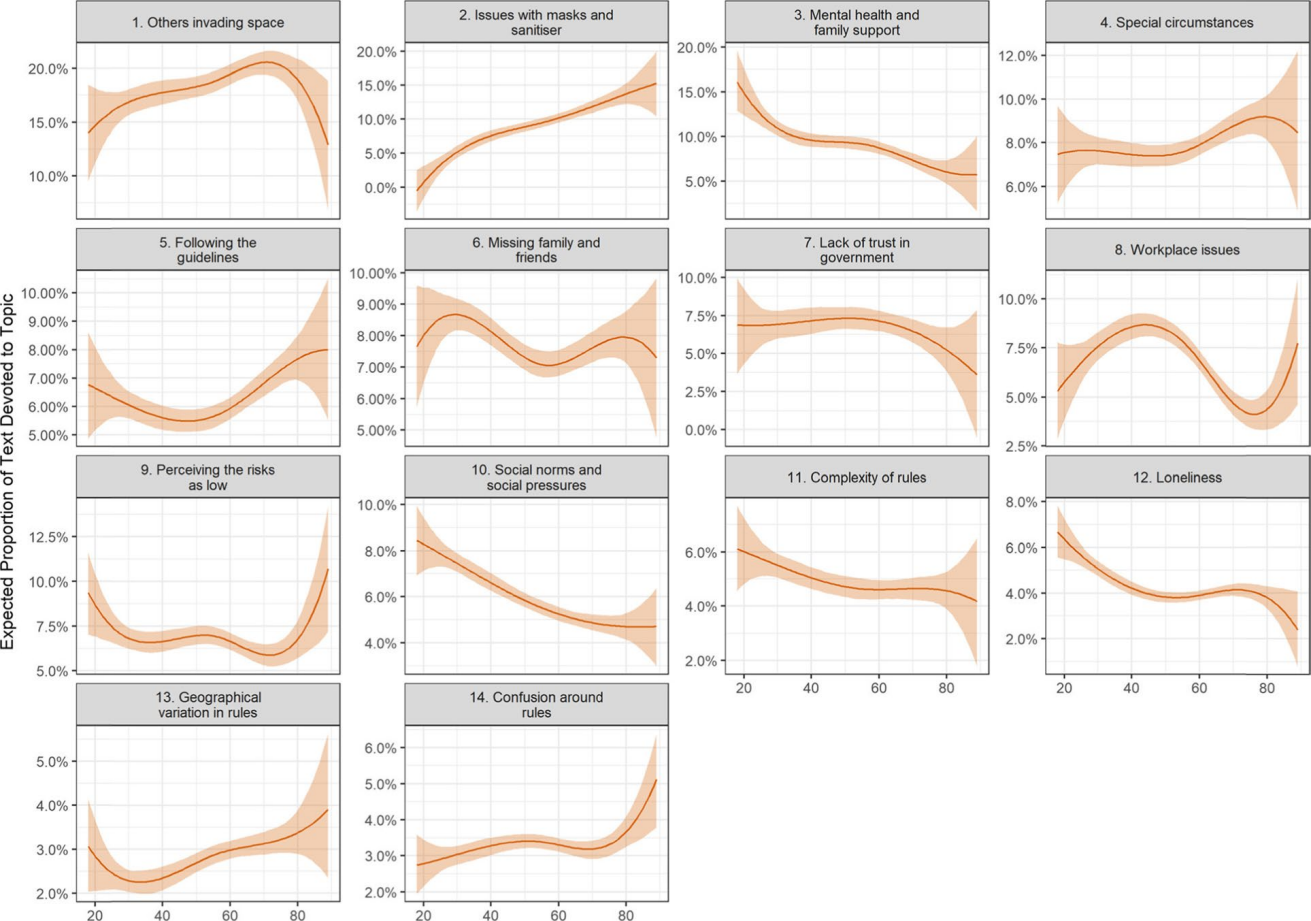


## Supplementary Information

**Additional file 1.** Supplementary file 1.
