# Supplementary material for: Correction to: Guidelines: a structural topic modelling analysis of free-text data from 17,500 UK adults
Source: BMC Public Health. 2022 Mar 24;22:581. doi: 10.1186/s12889-022-12614-1 (PMC8943932; doi:10.1186/s12889-022-12614-1)
Supplement: Supplementary file 1 — Additional file 1. Supplementary file 1. [file 12889_2022_12614_MOESM1_ESM.docx]

# Errors in Compliance facilitators and respondent characteristics

There were a number of differences according to age (Figure 2). Younger participants were more likely to discuss following the rules (Topic F4), working from home/support bubble (Topic F10), and protecting the NHS (Topic F13). Older people were more likely to discuss catching and transmitting COVID (Topic F1), protecting high risk and vulnerable (Topics F2 and F6), and consuming public information (Topic F3). Interestingly, there was a U-shaped association between age and Topic F11 (activities and Zoom), with younger and older people more likely to discuss the topic than the middle aged.

# Errors in Compliance Barriers and Respondent Characteristics

Figures 5-7 display the results of models regressing topic proportions on respondent characteristics. There were a number of differences according to age (Figure 5). Older participants were more likely to discuss issues with masks and sanitiser (Topic B2) and geographic variation in rules (Topic B13) and confusion around rules (Topic B14). Young people were more likely to mention mental health and family support (Topic B3), social pressures (Topic B10), and loneliness and social isolation (Topic B12).

# Errors in Discussion

The topics that participants discussed were related to participant characteristics. Notably, younger individuals were more likely to mention social pressures as a barrier to compliance, and older individuals were more likely to discuss confusion around rules.
